# Impact of Colchicine Therapy on Ventriculoarterial Coupling in Familial Mediterranean Fever: A Cross-Sectional Study

**DOI:** 10.3390/jcm14196902

**Published:** 2025-09-29

**Authors:** Hakan Duman, Hüseyin Durak, Osman Cüre, Mustafa Çetin, Ali Gökhan Özyıldız, Elif Ergül, Müjgan Ayşenur Şahin, Ahmet Özsipahi, Ahmet Yasin Tuncer, Barış Dindar, Nadir Emlek

**Affiliations:** 1 Department of Cardiology, Faculty of Medicine, Recep Tayyip Erdoğan University, Rize 53020, Türkiye; 2 Department of Rheumatology, Faculty of Medicine, Recep Tayyip Erdoğan University, Rize 53020, Türkiye; 3 Department of Cardiology, Faculty of Medicine, Uşak University, Uşak 64000, Türkiye

**Keywords:** familial mediterranean fever, colchicine, ventriculo-arterial coupling, Ea/Es ratio, arterial stiffness, cardiovascular risk

## Abstract

**Background:** Familial Mediterranean Fever (FMF) is a chronic autoinflammatory disorder that is characterized by increased arterial stiffness and subtle cardiovascular dysfunction. Colchicine remains the mainstay of treatment and may provide vascular benefits that extend beyond its anti-inflammatory effects. However, the association between colchicine therapy and ventriculoarterial coupling (VAC), a hemodynamic marker of cardiovascular efficiency, has not been previously studied. **Methods:** In this cross-sectional study, 97 patients with FMF receiving colchicine therapy for at least one year and 81 colchicine-naive individuals without FMF were consecutively enrolled from a tertiary rheumatology outpatient clinic. The VAC was evaluated using the Chen method, calculated as the ratio of arterial elastance (Ea) to end-systolic elastance (Es), based on echocardiographic measurements and noninvasive brachial blood pressure. Correlation analyses and stepwise multivariate linear regression analyses were performed to identify independent predictors of VAC. **Results:** Patients with FMF demonstrated significantly lower VAC values compared to controls (1.23 ± 0.34 vs. 1.40 ± 0.57; *p* = 0.001). The colchicine dose was inversely correlated with VAC (r = −0.243; *p* = 0.001) and remained an independent predictor in multivariate analysis (β = −0.186, *p* = 0.018). Beta-blocker use was positively associated with VAC (β = 0.194, *p* = 0.014), whereas female sex showed a borderline inverse association. **Conclusions:** Colchicine use in patients with FMF was associated with more favorable VAC in a dose-dependent manner. These findings suggest that colchicine may exert cardiovascular effects beyond the control of inflammation. VAC may be a useful noninvasive marker for assessing vascular–ventricular interactions in FMF.

## 1. Introduction

Familial Mediterranean Fever (FMF) is the most prevalent monogenic autoinflammatory disorder, primarily affecting populations of Mediterranean origin [1]. It is clinically characterized by recurrent episodes of fever, serositis, and elevated levels of acute-phase reactants. Increasing evidence suggests that FMF should be regarded as a systemic inflammatory condition with multisystemic implications including vascular involvement [2]. Although colchicine remains the cornerstone of therapy, effectively preventing acute attacks and the development of amyloidosis, recent studies have indicated that subclinical inflammation may persist during clinically quiescent periods and contribute to endothelial dysfunction and increased cardiovascular risk [3].

Chronic low-grade inflammation, a defining feature of FMF, has been implicated in early vascular aging, increased arterial stiffness, and subtle myocardial dysfunction [4]. Previous studies have reported impairments in left ventricular performance and vascular compliance in patients with FMF, even in the absence of clinically overt cardiovascular disease [2]. However, the underlying pathophysiological mechanisms and functional markers of cardiovascular involvement in FMF have yet to be fully elucidated.

Colchicine, a microtubule polymerization inhibitor, exerts anti-inflammatory effects by suppressing neutrophil activity, inflammasome assembly, and cytokine release. Beyond its role in autoinflammatory disorders, colchicine has shown promising results in reducing cardiovascular events in coronary artery disease and pericarditis [5]. These effects are thought to arise from the modulation of endothelial function, vascular smooth muscle tone, and oxidative stress [6]. However, the net cardiovascular impact of long-term colchicine use in non-atherosclerotic populations, such as those with FMF, remains unclear.

Ventriculoarterial coupling (VAC), defined as the ratio of arterial elastance (Ea) to end-systolic elastance (Es), represents an integrated measure of cardiovascular performance that incorporates both left ventricular contractility and arterial load. It can be assessed noninvasively using echocardiographic imaging and brachial blood pressure measurements. VAC offers a physiological perspective on myocardial–vascular interaction that extends beyond conventional metrics such as ejection fraction or blood pressure alone [7,8]. In contrast to isolated metrics, such as ventricular function or pulse wave velocity, VAC offers a comprehensive assessment of cardiovascular energy transfer and pump function. It provides system-level insights into cardiac performance, which are particularly pertinent in inflammatory conditions in which both ventricular and arterial properties may be compromised [9]. Elevated VAC values indicate ventricular–arterial mismatch, whereas lower values may reflect more efficient coupling or, in some cases, reduced ventricular elastance [10]. Alterations in VAC have been linked to adverse clinical outcomes in patients with heart failure and hypertension [7]. In the context of FMF, where chronic inflammation may lead to subclinical cardiovascular dysfunction affecting both ventricular mechanics and arterial properties, VAC represents a comprehensive assessment tool that can detect early cardiovascular inefficiency before overt clinical manifestations [11].

Although increased arterial stiffness has been consistently reported in patients with FMF and appears to improve with colchicine therapy, VAC, a more integrative marker of cardiovascular performance, has been scarcely investigated in this context. Emerging evidence suggests that colchicine resistance or subtherapeutic dosing may contribute to persistent vascular dysfunction and residual cardiovascular risk, even during clinical remission [12]. A deeper understanding of the influence of colchicine on the VAC could help uncover cardiovascular effects that extend beyond inflammation control. Beyond its established role in FMF, colchicine has recently gained attention for its cardiovascular implications, including potential benefits in coronary artery disease and other vascular conditions [13]. However, it remains unclear whether long-term colchicine therapy alters VAC primarily through anti-inflammatory mechanisms or exerts dose-dependent effects on the ventricular and vascular dynamics. In this context, the present study aimed to evaluate VAC in patients with FMF receiving stable colchicine therapy and to examine its association with colchicine dose and other relevant clinical variables.

## 2. Methods

### 2.1. Study Design and Population

This cross-sectional study was conducted at a tertiary care rheumatology outpatient clinic between January 2024 and March 2025. A total of 178 adult participants (aged >18 years) were consecutively enrolled. We included 97 patients diagnosed with FMF according to Tel Hashomer criteria, all of whom had been receiving colchicine for at least one year. As a comparison group, 81 individuals without FMF and with no history of colchicine use were consecutively enrolled.

Patients were excluded if they had moderate to severe valvular heart disease, reduced left ventricular ejection fraction (LVEF < 50%), a history of cardiac surgery, arrhythmias, acute coronary syndrome, myocarditis, cardiomyopathy, secondary hypertension, stroke, endocrine disorders, electrolyte abnormalities, anemia, pulmonary thromboembolism, malignancy, advanced chronic kidney disease (estimated glomerular filtration rate [eGFR] < 30 mL/min/1.73 m^2^), or any active infection.

The study protocol was approved by the Ethics Committee of Recep Tayyip Erdoğan University. All the procedures were conducted in accordance with the principles outlined in the Declaration of Helsinki. Written informed consent was obtained from all the participants prior to their inclusion in the study.

### 2.2. Blood Pressure and Biochemical Measurements

Systolic blood pressure (SBP) was measured in the seated position after a 5-min rest using an automated oscillometric device (Omron M2; Omron Healthcare, Kyoto, Japan). Venous blood samples were drawn in the morning following an overnight fast to evaluate routine biochemical parameters, including serum creatinine, glucose, C-reactive protein (CRP), hemoglobin, albumin, and total protein. The eGFR was calculated using the CKD-EPI formula.

The colchicine dosage was recorded in mg per day. Data on comorbid conditions, including hypertension, diabetes mellitus, and hyperlipidemia, as well as smoking status, duration of FMF, and use of cardiovascular medications (beta-blockers, ACE inhibitors, angiotensin receptor blockers [ARBs], diuretics, and statins) were obtained from medical records and patient interviews.

### 2.3. Echocardiographic Evaluation and VAC Calculation

Transthoracic echocardiography was performed using a Vivid E95 system (Ultra Edition; GE Healthcare, Horten, Norway), equipped with an M5Sc transducer. All examinations were performed by a single experienced cardiologist with a minimum of five years of expertise in echocardiographic imaging.

Stroke volume (SV) and end-systolic volume (ESV) were calculated using either the modified biplane Simpson method or three-dimensional (3D) volumetric analysis, depending on image quality. VAC was calculated using the single-beat method proposed by Chen et al. as the ratio of arterial elastance (Ea) to end-systolic elastance (Es) [14]:

Ea = 0.9 × SBP/SV

Es = 0.9 × SBP/ESV

VAC = Ea/Es

All VAC values were expressed as unitless ratios.

### 2.4. Statistical Analysis

Continuous variables were presented as mean ± standard deviation (SD), while categorical variables were summarized as counts and percentages. Depending on the data distribution, Pearson or Spearman correlation coefficients were used to examine the associations between VAC and clinical or laboratory parameters.

Stepwise multivariate linear regression analysis was performed to identify independent predictors of VAC. Variables with a *p*-value < 0.1 in univariate analyses were entered into the regression models. Statistical significance was set at a two-tailed *p*-value of <0.05. All analyses were conducted using IBM SPSS Statistics (version 19.0; SPSS Inc., Chicago, IL, USA).

## 3. Results

A total of 178 patients were included in the analysis, comprising 97 individuals (54.5%) with FMF and 81 colchicine-naive controls. The mean age of the study population was 38.9 ± 12.5 years, and 46.6% were female. Baseline characteristics of the study population are summarized in Table 1. Patients with FMF (*n* = 97) were significantly younger than controls (35.1 ± 12.3 vs. 43.6 ± 11.3 years, *p* < 0.001) and had lower BMI values (26.2 ± 4.8 vs. 28.3 ± 5.5 kg/m^2^, *p* = 0.005). The prevalence of diabetes mellitus (2.1% vs. 19.8%, *p* < 0.001), hypertension (6.2% vs. 21.0%, *p* = 0.003), hyperlipidemia (26.8% vs. 55.6%, *p* < 0.001), and coronary artery disease (0% vs. 13.8%, *p* < 0.001) was lower in FMF patients compared to controls. Similarly, the use of cardiometabolic medications—including β-blockers (0% vs. 23.5%, *p* < 0.001), ACE inhibitors/ARBs (4.1% vs. 17.3%, *p* = 0.004), diuretics (0% vs. 9.9%, *p* = 0.002), statins (0% vs. 7.4%, *p* = 0.008), and oral antidiabetics (0% vs. 7.4%, *p* = 0.008)—was significantly less frequent in FMF patients. Smoking was also less prevalent in FMF patients (20.6% vs. 36.7%, *p* = 0.014). Laboratory parameters showed higher eGFR (109.4 ± 17.9 vs. 102.2 ± 17.5 mL/min/1.73 m^2^, *p* = 0.003) and lower hemoglobin levels (13.4 ± 1.7 vs. 13.9 ± 1.8 g/dL, *p* = 0.046) in the FMF group.

Echocardiographic evaluation revealed that FMF patients had significantly lower VAC values compared to controls (1.23 ± 0.34 vs. 1.40 ± 0.57, *p* = 0.001). In addition, LVEF was reduced in FMF patients (60.2 ± 5.6 vs. 64.9 ± 12.3%, *p* < 0.001), despite being within the normal range in both groups.

**Figure 1:**
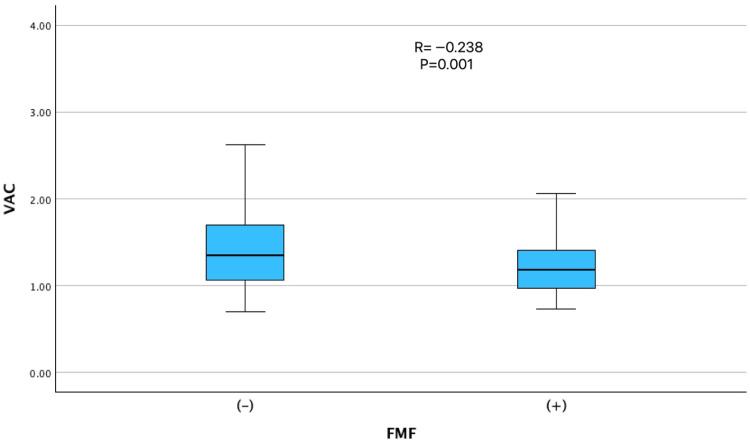
Comparison of ventriculo-arterial coupling (VAC) between patients with and without FMF. VAC was significantly lower in patients with FMF compared to non-FMF individuals (r = −0.238, *p* = 0.001), possibly reflecting an alteration in myocardial-vascular interaction related to FMF status.

**Figure 2:**
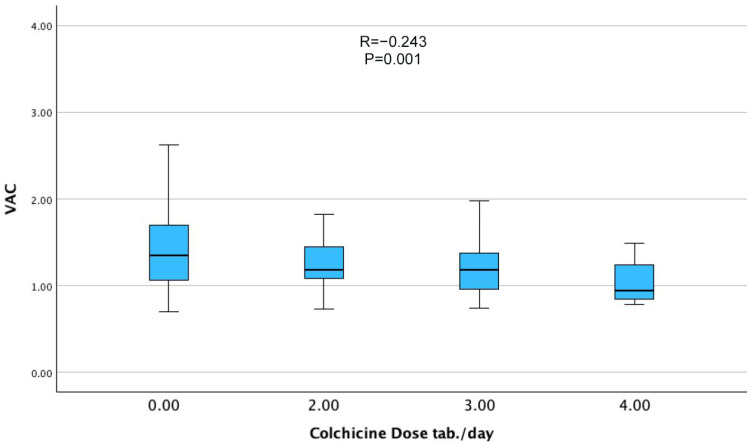
Dose-dependent relationship between colchicine intake and ventriculo-arterial coupling (VAC). Increasing daily colchicine dose was associated with a gradual decline in VAC values (r = −0.243, *p* = 0.001), which may reflect a dose-related influence on ventricular-vascular interaction.

Correlation analysis (Table 2) demonstrated that VAC was inversely associated with FMF diagnosis (r = −0.238, *p* = 0.001) (Figure 1) and colchicine dose (r = −0.243, *p* = 0.001), demonstrating a clear dose-response relationship across increasing levels of colchicine intake (Figure 2). Positive correlations were observed with age (r = 0.196, *p* = 0.009), hypertension (r = 0.162, *p* = 0.032), and hyperlipidemia (r = 0.167, *p* = 0.025). Notably, LVEF was not significantly correlated with VAC, underscoring that VAC provides complementary information beyond global systolic function.

**Figure 3:**
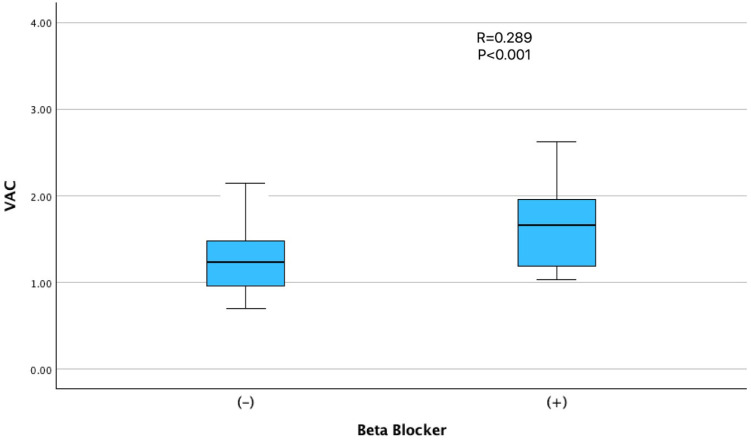
Relationship between beta-blocker use and ventriculoarterial coupling (VAC). Patients using beta-blockers exhibited significantly higher VAC values than non-users (r = 0.289, *p* < 0.001).

Among the medications evaluated, beta-blocker use showed the strongest positive correlation with VAC (r = 0.289; *p* < 0.001), which is consistent with the known hemodynamic effects of this drug class on arterial compliance and afterload reduction (Figure 3). The use of ACE inhibitors or ARBs (r = 0.217; *p* = 0.004) and diuretics (r = 0.188; *p* = 0.012) was also positively associated with VAC (Table 2). No significant correlations were observed between VAC and smoking status, CRP levels, FMF disease duration or severity/activity scores.

In multivariate linear regression models, both FMF diagnosis (β = −0.176; *p* = 0.027) and colchicine dosage (β = −0.186; *p* = 0.018) emerged as independent predictors of lower VAC values. Beta-blocker use remained positively associated with VAC across all models (β = 0.194–0.197; *p* < 0.05), while female sex demonstrated a borderline inverse association in both models (Table 3).

## 4. Discussion

Consistent with our hypothesis, long-term colchicine therapy in patients with FMF was associated with altered VAC values in this study. These results highlight the potential role of colchicine in modulating ventricular–vascular interactions. Importantly, the observed inverse relationship between colchicine dose and VAC suggests a dose-related trend that, although not establishing causality, provides novel insights into the possible cardiovascular implications of colchicine therapy. To our knowledge, this is the first study to evaluate VAC in FMF using noninvasive echocardiographic methods, thereby extending our current understanding of the cardiovascular effects of colchicine beyond inflammation control.

Although various indices have been utilized to assess ventricular performance and arterial stiffness separately, VAC was selected in this study because it provides an integrative measure that simultaneously reflects ventricular contractility and arterial load. Unlike conventional echocardiographic parameters, VAC provides a more comprehensive assessment of cardiovascular efficiency and has been associated with clinical outcomes in both heart failure with preserved ejection fraction (HFpEF) and hypertensive heart disease [15]. Deviations from the optimal VAC range, whether toward excessive or insufficient coupling, are indicative of impaired cardiovascular performance and increased energetic costs for a given cardiac output [16]. In this context, the observed reduction in VAC among patients with FMF may reflect two possible interpretations: a more efficient ventricular–arterial interaction mediated by colchicine’s anti-inflammatory and vascular-stabilizing effects, or an early manifestation of subclinical ventricular dysfunction related to chronic inflammation. Previous studies have consistently reported increased arterial stiffness in FMF, largely attributed to sustained low-grade inflammation and endothelial dysfunction [2,4]. Our findings may therefore appear paradoxical. This apparent discrepancy might be reconciled by considering that lower VAC values, although generally interpreted as reflecting more favorable efficiency, could also arise from reduced ventricular elastance in the setting of chronic inflammation. Thus, the physiological meaning of lower VAC in FMF requires cautious interpretation, and further validation through longitudinal studies and mechanistic assessments is warranted. Importantly, this perspective is in line with the established vascular-stabilizing properties of colchicine, which may partly account for the paradoxical findings. The improvement in VAC following colchicine therapy in patients with FMF may translate into clinically meaningful benefits through enhanced cardiovascular efficiency and reduced long-term risk of cardiovascular events, particularly given the established cardioprotective effects of colchicine in other inflammatory conditions and its demonstrated ability to improve arterial stiffness parameters in FMF [17]. Although LVEF was lower in FMF patients than in controls, LVEF was not significantly correlated with VAC, indicating that VAC captures aspects of ventricular–arterial interaction that are not reflected by global systolic function and thereby provides complementary information to conventional indices.

Colchicine exerts cardiovascular protective effects in various clinical settings, including post–myocardial infarction and chronic coronary syndromes, primarily through inhibition of the NLRP3 inflammasome, IL-1β signaling, and leukocyte trafficking [18]. The observed inverse association between colchicine dosage and VAC may suggest potential mechanistic associations, although causality cannot be inferred due to the cross-sectional design. The anti-inflammatory properties of colchicine may attenuate vascular stiffness and enhance endothelial function, thereby reducing arterial elastance [19]. In contrast, colchicine disrupts microtubule dynamics, which are essential for maintaining myocardial cytoskeletal architecture and calcium homeostasis [20]. In our cohort, higher colchicine doses independently predicted more favorable VAC values, suggesting a potential vascular-modulatory effect that extends beyond inflammation control. However, at higher doses, colchicine may also reduce myocardial contractility and end-systolic elastance, potentially contributing to lower VAC values. These proposed mechanisms remain hypothetical, as we did not directly assess endothelial function, myocardial contractility, or related biomarkers. Therefore, further mechanistic studies are needed to validate these interpretations. While colchicine resistance and subtherapeutic dosing remain relevant challenges in FMF management, our findings suggest that effective colchicine dosing may influence ventriculoarterial interactions and possibly reduce long-term cardiovascular risk. Notably, this association remained significant after adjustment for age, sex, and use of antihypertensive medications in the multivariate models, suggesting an independent pharmacological effect.

Previous studies have demonstrated subclinical myocardial dysfunction in patients with FMF, particularly during attack-free periods, which is often accompanied by elevated inflammatory markers and increased arterial stiffness [4,21]. However, most of these investigations did not include integrative hemodynamic parameters such as VAC. By showing a dose-dependent relationship between colchicine use and VAC, our study offers a novel perspective on the cardiovascular effects of colchicine that extends beyond its established anti-inflammatory properties.

In our study, β-blocker use was positively associated with VAC values, which at first may seem counterintuitive. However, β-blockers are known to reduce heart rate, decrease myocardial oxygen demand, and improve arterial compliance, all of which may favorably influence ventriculo-arterial interactions by lowering ventricular afterload. Prior studies have similarly demonstrated improvements in VAC with β-blocker therapy, particularly in patients with heart failure [22]. Nevertheless, the number of β-blocker users in our cohort was relatively small, and indication bias cannot be excluded. Therefore, this association should be interpreted with caution. Additionally, the borderline inverse association between female sex and VAC is also in line with established sex-related differences in vascular architecture and myocardial stiffness, which are thought to be influenced by hormonal and autonomic factors [23].

Our findings have several clinical implications. First, VAC may represent a sensitive and noninvasive marker for detecting early cardiovascular changes in patients with FMF, enabling risk stratification, even in asymptomatic individuals. Second, the apparent dose-dependent effect of colchicine on VAC underscores the importance of personalized dosing strategies, particularly for patients with concurrent cardiovascular risk factors. The trends illustrated in Figure 1, Figure 2 and Figure 3 further support the interpretation that FMF status, colchicine dosage, and beta-blocker therapy were independently associated with VAC physiology. Whether reduced VAC values under colchicine therapy reflect enhanced cardiovascular efficiency or early subclinical dysfunction remains uncertain and warrants investigation in future longitudinal studies employing advanced imaging techniques and functional assessments, such as exercise testing.

## 5. Limitations

This study had several limitations. Its cross-sectional design limits its ability to draw causal inferences regarding the relationship between colchicine use and VAC. We also acknowledge that all FMF patients in our cohort were receiving colchicine therapy; therefore, the absence of an untreated FMF group (e.g., newly diagnosed or colchicine-resistant patients) limits our ability to distinguish between disease-related effects and treatment-related effects.

An important consideration in interpreting our findings is that the control group was older and exhibited a higher prevalence of cardiometabolic risk factors, including hypertension, diabetes mellitus, hyperlipidemia, and coronary artery disease. Since both age and these comorbidities are known to adversely affect vascular function and ventricular–arterial interaction, baseline imbalances may have contributed to the observed differences in VAC between groups. Although multivariable analysis was performed to adjust for these potential confounders, residual confounding cannot be excluded. Interestingly, FMF patients were not only younger but also displayed a more favorable cardiovascular risk profile, which may partly reflect the cumulative benefits of long-term colchicine therapy through its anti-inflammatory and vascular-stabilizing effects. This hypothesis, however, remains speculative and requires confirmation in prospective studies specifically designed to evaluate the long-term cardioprotective role of colchicine in FMF.

Although echocardiographic assessments were standardized and conducted by a single experienced operator to minimize variability, invasive hemodynamic elastance measurements were not obtained. Furthermore, as all echocardiographic measurements were performed by a single operator, inter-observer variability could not be assessed. Intra-observer variability was not formally evaluated, representing an additional limitation of our study.

We did not evaluate inflammatory markers or endothelial function in any of the participants, which may have offered additional mechanistic insight.

Finally, we acknowledge that the single-center design and modest sample size may restrict the generalizability of our findings. Future multicenter studies with larger cohorts are warranted to validate these results.

## 6. Conclusions

In conclusion, our findings suggest a possible association between colchicine therapy and altered VAC in patients with FMF, with higher colchicine doses related to lower VAC values. Prospective studies are needed to clarify the cardiovascular implications of colchicine therapy and the hemodynamic effects of chronic inflammation in autoinflammatory conditions.

## Figures and Tables

**Table 1:** Baseline Characteristics of the Study Population.

Variable	FMF (−) (n = 81)	FMF (+) (n = 97)	*p*-Value
Age, years	43.6 ± 11.3	35.1 ± 12.3	<0.001
Female sex, n (%)	38 (46.9)	45 (46.4)	0.532
Body mass index, kg/m^2^	28.3 ± 5.5	26.2 ± 4.8	0.005
Disease duration, years	0 (0)	13.9 ± 10.1	<0.001
Diabetes mellitus, n (%)	16 (19.8)	2 (2.1)	<0.001
Hypertension, n (%)	17 (21.0)	6 (6.2)	0.003
Hyperlipidemia, n (%)	45 (55.6)	26 (26.8)	<0.001
Coronary artery disease, n (%)	11 (13.8)	0 (0.0)	<0.001
Smoking, n (%)	29 (36.7)	20 (20.6)	0.014
β-blocker use, n (%)	19 (23.5)	0 (0.0)	<0.001
CCB use, n (%)	9 (11.1)	4 (4.1)	0.067
ACE inhibitor or ARB, n (%)	14 (17.3)	4 (4.1)	0.004
Diuretic use, n (%)	8 (9.9)	0 (0.0)	0.002
Statin use, n (%)	6 (7.4)	0 (0.0)	0.008
Oral antidiabetic use, n (%)	6 (7.4)	0 (0.0)	0.008
WBC (×10^3^/µL)	7.5 ± 1.9	7.3 ± 2.0	0.538
Hemoglobin, g/dL	13.9 ± 1.8	13.4 ± 1.7	0.046
eGFR, mL/min/1.73 m^2^	102.2 ± 17.5	109.4 ± 17.9	0.003
Serum creatinine, mg/dL	0.79 ± 0.18	0.73 ± 0.17	0.053
Fasting glucose, mg/dL	102.4 ± 32.4	90.3 ± 9.3	<0.001
VAC (Ea/Es ratio)	1.40 ± 0.57	1.23 ± 0.34	0.001
LVEF, %	64.9 ± 12.3	60.2 ± 5.6	<0.001

Abbreviations: ACE: angiotensin-converting enzyme; ARB: angiotensin receptor blocker; CCB: calcium channel blocker; eGFR: estimated glomerular filtration rate; LVEF: left ventricular ejection fraction; VAC: ventriculo-arterial coupling; WBC: white blood cell.

**Table 2:** Correlation Between Clinical Variables and VAC (Ea/Es Ratio).

Variable	r	*p*-Value
Age, years	0.196	0.009
Female sex	−0.162	0.031
FMF diagnosis	−0.238	0.001
FMF severity/activity scores	NS	NS
Disease duration, years	NS	NS
Body mass index, kg/m^2^	NS	NS
Diabetes mellitus	NS	NS
Hypertension	0.162	0.032
Hyperlipidemia	0.167	0.025
Coronary artery disease	NS	NS
Smoking	NS	NS
β-blocker use	0.289	<0.001
CCB use	NS	NS
ACE inhibitor/ARB use	0.217	0.004
Diuretic use	0.188	0.012
Statin use	NS	NS
Colchicine dose, mg/day	−0.243	0.001
WBC (×10^3^/µL)	NS	NS
Lymphocyte count (×10^3^/µL)	0.188	0.012
Total protein, g/dL	NS	NS
Albumin, g/dL	NS	NS
Hemoglobin, g/dL	NS	NS
Fasting glucose, mg/dL	NS	NS
Serum creatinine, mg/dL	NS	NS
eGFR, mL/min/1.73 m^2^	−0.142	0.063
C-reactive protein, mg/L	NS	NS
LVEF, %	NS	NS
IVS thickness, mm	NS	NS
PW thickness, mm	NS	NS
E/A ratio	NS	NS
E/e′ ratio	NS	NS

Abbreviations: ACE: angiotensin-converting enzyme; ARB: angiotensin receptor blocker; CCB: calcium channel blocker; eGFR: estimated glomerular filtration rate; LVEF: left ventricular ejection fraction; VAC: ventriculo-arterial coupling; WBC: white blood cell. Note: NS = not significant. Correlation coefficients are Pearson or Spearman, depending on variable distribution.

**Table 3:** Multivariate Linear Regression Models for Predictors of VAC.

Model 1				
Variable	Unstandardized B	Standardized β	95% CI	*p*-Value
FMF diagnosis	−0.171	−0.176	−0.322 to −0.020	0.027
β-blocker use	0.304	0.197	0.060 to 0.548	0.015
Female sex	−0.123	−0.127	−0.264 to 0.018	0.088
**Model 2**				
**Variable**	**Unstandardized B**	**Standardized β**	**95% CI**	***p*-Value**
Colchicine dose	−0.063	−0.186	−0.115 to −0.011	0.018
β-blocker use	0.301	0.194	0.058 to 0.543	0.014
Female sex	−0.216	−0.130	−0.267 to 0.015	0.079

Abbreviations: CI: confidence interval; FMF: familial mediterranean fever; VAC: ventriculo-arterial coupling. Note: Model 1 includes FMF diagnosis. Model 2 includes colchicine dose instead of FMF diagnosis.

## Data Availability

The data presented in this study are available upon reasonable request from the corresponding author.
